# Next-generation sequencing for identification of actionable gene mutations in intestinal-type sinonasal adenocarcinoma

**DOI:** 10.1038/s41598-020-80242-z

**Published:** 2021-01-26

**Authors:** Paula Sánchez-Fernández, Cristina Riobello, María Costales, Blanca Vivanco, Virginia N. Cabal, Rocío García-Marín, Laura Suárez-Fernández, Fernando López, Rubén Cabanillas, Mario A. Hermsen, José Luis Llorente

**Affiliations:** 1 Department Otolaryngology, Hospital Universitario Central de Asturias, Oviedo, Spain; 2 Department Head and Neck Oncology, Instituto de Investigación Sanitaria del Principado de Asturias (ISPA), Instituto Universitario de Oncología del Principado de Asturias (IUOPA), Centro de Investigación Biomédica en Red (CIBER-ONC), Edf. FINBA, N-1 F49, C/ Avenida de Roma s/n, 33011 Oviedo, Spain; 3 Department Pathology, Hospital Universitario Central de Asturias, Oviedo, Spain; 4 Cabanillas Precision Consulting, Zurich, Switzerland

**Keywords:** Head and neck cancer, Molecular medicine

## Abstract

Intestinal-type sinonasal adenocarcinoma (ITAC) is a rare tumor carrying poor prognosis and needing new treatment options. The aim of this study was to identify actionable gene mutations that can guide new personalized target-specific therapies in ITAC patients. A series of 48 tumor and 27 corresponding germline DNA samples were analyzed by next generation sequencing using a panel of 120 genes. In total, 223 sequence variants were found in 70 genes. Matched tumor/germline comparison in 27 cases revealed that 57% were in fact germline variants. In 20 of these 27 cases, 58 somatic variants in 33 different genes were identified, the most frequent being PIK3CA (5 cases), APC and ATM (4 cases), and KRAS, NF1, LRP1B and BRCA1 (3 cases). Many of the somatic gene variants affected PI3K, MAPK/ERK, WNT and DNA repair signaling pathways, although not in a mutually exclusive manner. None of the alterations were related to histological ITAC subtype, tumor stage or survival. Our data showed that thorough interpretation of somatic mutations requires sequencing analysis of the corresponding germline DNA. Potentially actionable somatic mutations were found in 20 of 27 cases, 8 of which being biomarkers of FDA-approved targeted therapies. Our data implicate new possibilities for personalized treatment of ITAC patients.

## Introduction

Sinonasal intestinal-type adenocarcinoma (ITAC) is a relatively rare tumor, etiologically related to occupational exposure to wood and leather dust^1–6^. Chronic inflammation resulting from wood dust exposure may cause genetic and epigenetic changes that promote proliferation and growth. Supporting this hypothesis, cyclooxygenase-2 (COX-2) expression has been correlated to wood dust etiology^7^ and G > A missense mutations in TP53 and KRAS, supposedly caused by free radical species released by leukocytes in a chronic inflammatory environment, have been found twice as frequent as tobacco-associated G > T mutations^8,9^. The World Health Organization classification of head and neck tumors distinguishes papillary, colonic, solid, and mucinous type, the latter two having worse clinical behavior^1^. In spite of advances in imaging, endoscopic surgical techniques and precision radiotherapy^2,10^, patients with ITAC still face an unfavorable prognosis, with a 5-year survival rate ranging from 35% for stage IV to 80% for stage I tumors. Local recurrence often occurs within two years of follow-up and is the main contributor to sinonasal cancer mortality^1,2,6,10–17^. Therefore, there is a need for alternative drugs for neoadjuvant, concomitant o adjuvant therapy in ITAC.

Molecular-genetic characteristics are more and more used as indicators for treatment with modern specific small molecule or monoclonal antibody inhibitors in many tumor types including sinonasal carcinomas^3^. Multiplex ligation-dependent probe amplification (MLPA) and comparative genomic hybridization (CGH) analysis revealed frequent gains on chromosome arms 5p, 7q, 8q, 12p, and 20q and losses on 4q, 5q, 8p, 17p, and 18q. Gains on 5p, 12p, and losses at 4q are typical of ITAC and have not been reported in other adenocarcinomas^18–21^. Using cluster analysis based on gene copy number alterations, López-Hernández et al. presented five ITAC subgroups with distinct clinical outcomes^22^. Studies on specific genes have been guided by results from other adenocarcinomas, particularly colorectal and lung, and revealed 40%-50% TP53 mutations^8,23,24^, approximately 15% KRAS mutations and absence of mutations in EGFR, BRAF^9,22,25–29^, APC and CTTNB1^29,30^. Protein overexpression of tyrosine receptor kinases EGFR, MET and FGFR1 have also been reported in ITAC^27,31–33^. Nuclear b-catenin expression was observed in approximately 30%–50% of cases^29,34,35^.

In contrast to other cancer types where several activating mutations with therapeutic implications have been described^36^, few clinically relevant genetic data on ITAC have been published thus far. Functional p53 has been correlated to better outcome in patients treated with induction chemotherapy and could be used as predictor^37^. More such genetic studies are needed to develop new options for treatment of ITAC. Every year, new specific small molecule or monoclonal antibody inhibitors are being developed and become available for oncological treatment. A number are already clinically tested and in use in more frequent cancer types, others are in preclinical development. These modern drugs may also be effective in ITAC if the same genes or signaling pathways are affected.

The aim of this retrospective study was to evaluate the feasibility of mutation analysis by sequencing a panel of 120 actionable genes for identification of molecular targets that could guide the choice for personalized treatment of patients with ITAC.

## Material and methods

### Sample selection and clinical variables

Forty-eight fresh frozen samples of histopathologically confirmed ITAC, representative of this type of tumor in terms of distribution of disease stage and histological type, were collected from the biobank archives of our hospital. In 27 cases, also peripheral blood samples could be obtained. Written informed consent for the collection, storage, and analysis of specimens was obtained from all patients. All experimental protocols were approved by the Institutional Ethics Committee of the Hospital Universitario Central de Asturias and by the Regional CEIC from Principado de Asturias (approval numbers 66/15 for project PI15/01,629 and 83/17 for PI17/00,763). All methods were carried out in accordance with the guidelines of our institutional ethical committees (Comité de Ética de la Investigación del Principado de Asturias).

All patients were treated between 1998 and 2014 in the Otolaryngology department at the Hospital Universitario Central de Asturias (Oviedo, Spain); the clinical data are shown in Table 1. All patients were men and the mean age at diagnosis was 70 years (range 49–88 years). Forty-seven (98%) had an etiology of occupational exposure to wood dust. Twenty-five patients were tobacco smokers and 23 had never smoked. According to the 2017 World Health Organization histological classification^1^, 4 (8%) were papillary, 27 (56%) colonic, 6 (13%) solid, and 11 (23%) mucinous type ITAC. Ten cases (21%) were disease stage I, 9 (19%) stage II, 16 (33%) stage III, 6 (12%) stage IVa and 7 (15%) stage IVb. All 48 patients were treated by surgery, 18 by open and 30 by endoscopic approaches, while 25 patients (52%) received complementary radiotherapy and 1 patient (2%) received chemotherapy.

**Table 1:** Clinical features of 48 ITACs.

	No. patients (%)
**Sex**	
Women	0 (0)
Men	48 (100)
**Wood dust exposition**	
Yes	47 (98)
No	1 (2)
**Tobacco smoking**	
Yes	25 (52)
No	23 (48)
**Disease stage**	
I	10 (21)
II	9 (19)
III	16 (33)
IVa	6 (12)
IVb	7 (15)
**Orbit invasion**	
Periorbit	4 (8)
Orbit	2 (4)
No	44 (92)
**Intracranial invasion**	
Yes	4 (8)
No	44 (92)
**Histological subtype**	
Papillary	4 (8)
Colonic	27 (56)
Solid	6 (13)
Mucinous	11 (23)
**Complementary Radiotherapy**	
Yes	25 (52)
No	23 (48)
**Recurrence/Metastasis**	
Recurrence	30 (63)
Metastasis	8 (17)
None	18 (37)
**Patient status**	
Alive	12 (25)
Died of disease	23 (48)
Died other causes	13 (27)

### Panel design

Our selection of 120 actionable genes was made on the basis of the following criteria: 1) Could be directly targeted by an United States Food and Drug Administration (FDA)-approved drug; 2) Could be directly or indirectly targeted by a drug that is under investigation in clinical trials; 3) Could be directly or indirectly targeted by a drug under investigation in preclinical studies. Next-generation sequencing probes were designed to cover the whole coding sequence (i.e. all exons) of the following 120 genes: AKT, AKT1, AKT3, ALK, APC, AR, ARAF, ATM, ATR, AURKA, BAP1, BCL2L1, BCR-ABL1, BCR-JAK2, BRAF, BRCA1, BRCA2, BRD4, CBL, CCND1, CCNE1, CDK4, CDK6, CDKN1A, CDKN1B, CDKN2A, CDKN2B, CDKN2C, COL1A1-PDGFRB, CRLF2, CSF1R, CSF3R, CTNNB1, DDR2, DNMT3A, EGFR, EPHA2, ERBB2, ERBB3, ERBB4, ERCC1, ERS1, EZH2, FBW7, FBXW7, FGFR1, FGFR2, FGFR3, FLT3, FOXA1, FOXL2, FOXP1, GNA11, GNAQ, HGF, HRAS, IDH1, IDH2, IGF1, IGF1R, IGF2, IL10, IL7R, INPP4B, JAK1, JAK2, JAK3, KIT, KRAS, LRP1B, MAP2K1, MAP2K2, MAP2K4, MCL1, MDM2, MET, MGMT, MITF, MLL, MPL, mTOR, MYCN, MYD88, NF1, NF2, NFKB1, NFKB2, NOTCH1, NOTCH2, NOTCH3, NPM1, NRAS, NTRK1, PALB2, PDGFRA, PIK3CA, PIK3R1, PIK3R2,PML-RARA, PTCH1, PTEN, RAC1, RAF1, RB1, RET, RET-PTC1, ROS1, SH2B3, SMO, SOCS1, STAG2, STK11, TMPRSS2-ERG, TMPRSS2-ETV1, TSC1, TSC2.

### DNA extraction and next-generation sequencing

Genomic DNA was extracted from frozen tumor samples using the QIAGEN tissue extraction kit (QIAGEN GmbH, Hilden, Germany), and genomic DNA was extracted from blood samples by using High Pure PCR Template Preparation Kit (Roche Diagnostics GmbH, Mannheim, Germany). Next-generation sequencing (NGS) was performed using the SureSelect QXT Target Enrichment Kit for Illumina Multiplexed Sequencing (Agilent Technologies, Santa Clara CA, USA) following the manufacturer’s instructions (Protocol Version D0, November 2015). Twenty-five ng of genomic DNA quantified using Qubit HS dsDNA kit was fragmented and adaptors were added in a single enzymatic step. The adaptor-tagged DNA library was purified and amplified. Next, 750 ng of each library was hybridized using SureSelect QXT capture library (Agilent Technologies, Santa Clara CA, USA). The resulting libraries were recovered using Dynabeads MyOne Streptavidin T1 magnetic beads (Life Technologies, Madrid, Spain) and, a post-capture PCR amplification and indexing of the samples was carried out. After each step, the purification step was performed with AMPure XP beads (Beckman Coulter, Brea CA, USA) to remove short fragments such as adapter dimers. The quality of the libraries was assessed on a Bioanalyzer High Sensitivity DNA chip (Agilent Technologies, Santa Clara CA, USA). Based on DNA concentration and average fragment size, libraries were normalized to an equal concentration, 5 nM, and pooled by equal volume in 16-plex pools. Sequencing pools were then sequenced in a MiSeq system (Illumina Inc.) at the sequencing service of IMOMA (Oviedo, Spain). The average coverage of the sequencing was at least 500X readings for the tumor DNA samples and approximately 300X in the germline DNA (peripheral blood lymphocytes) samples.

### Bioinformatic analysis

All raw sequence data from the 48 ITAC tumor samples were processed using the bioinformatics software HD Genome One (DREAMgenics, Oviedo, Spain), certified with IVD/CE-marking (Supplementary Materials gives a comprehensive description of the analysis). The datasets generated in the study are in the process of depositing in a publicly available repository.

As starting point for the selection of genetic alterations with clinical relevance, we filtered out the sequence variants with a minor allele frequency > 1% in the normal population^38^. Next, we focused on non-synonymous changes with an impact on the sequence of the protein encoded by the gene and those that appear registered at COSMIC or ICGC databases^39^. With respect to the 21 tumor-only cases, we rejected variants with a coverage of less than 50 reads in order to avoid artifacts. For the filtering process of the 27 matched tumor/germline sequencing results the somatic status of sequence variants is unequivocal in itself^39^. For this reason, we have kept all variants with a minor allele frequency of up to 5% in the normal population without increasing the background noise^39,42^. Furthermore, we applied a cut-off keeping only the variants with an allelic frequency > 10% of the total reads in the tumor sample based on the rationale that clinically relevant driver mutations are expected to appear earlier in carcinogenesis and have a higher variant allele frequency than passenger alterations^40^. Nevertheless, due to tumor heterogeneity and admixture with normal cells (stroma, immune cells), we found lower frequencies for the heterozygous mutations than the theoretical 0.5 value of allele frequency expected in a pure clone^41^. Finally, we decided to take into account only copy number gains ≥ 6; the analysis algorithm is described in Supplementary methods.

### Statistical analysis

Possible correlations between mutations and clinical parameters were statistically analyzed by SPSS 15.0 software for Windows (SPSS, Chicago, IL), using the Pearson chi-square test, Fischer’s exact test, and Student’s t test. Kaplan–Meier analysis was performed for estimation of survival, comparing distributions of survival through the logarithmic range test (log-rank test). Values of P < 0.05 were considered significant.

## Results

### Patients follow-up

**Figure 1:**
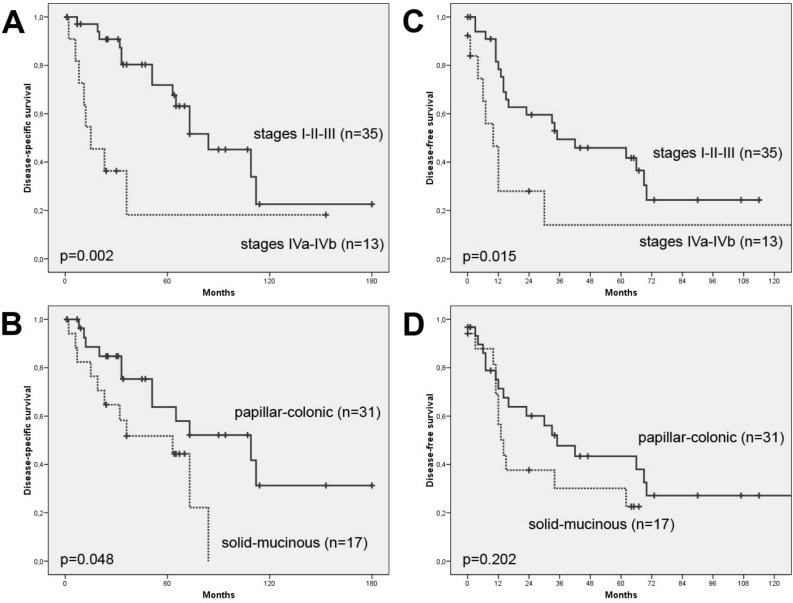
Disease-specific survival according to (**A**) tumor stage (I/II/III versus IVa/IVb) and to **B**) histological subtype (papilar/colonic versus solid/mucinous). Disease-free survival according to (**C**) tumor stage (I/II/III versus IVa/IVb) and to (**D**) histological subtype (papilar/colonic versus solid/mucinous).

During the period of follow-up (mean 49 months; range 1–180), 30 patients (63%) developed local recurrence, 8 (17%) of which together with distant metastasis. The median disease-free time was 30 months (range 1–153). At the time of writing, 12 of 48 patients (25%) remained disease-free and 23 of 48 (48%) died of recurrence or metastasis. Thirteen patients died during the postoperative period or due to intercurrent causes (Table 1). The disease-specific 5-year survival was 59%, and significantly correlated to tumor stage (log-rank 9.213, p = 0.002) and histological subtype (log-rank 3.911, p = 0.048). Tumor stage I/II cases showed longer disease-free survival compared to stage III/IV (Log rank 5.877, p = 0.015). A moderately longer disease-free survival was also observed in papillary and colonic compared to solid and mucinous subtypes ITAC (Log rank 1.607, p = 0.205) (Fig. 1).

### Panel sequencing

**Figure 2:**
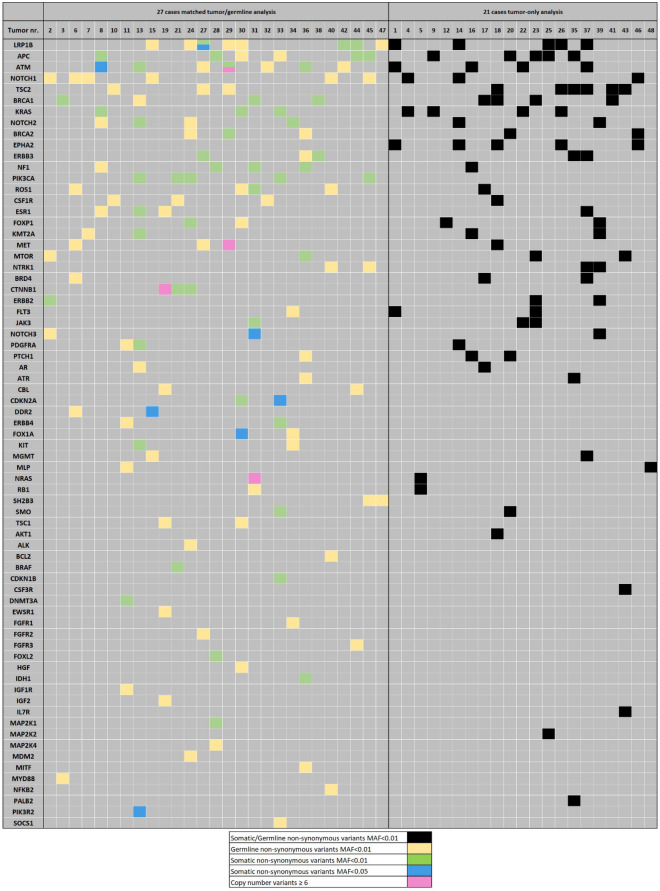
Sequence variants identified in 48 ITACs, 27 by tumor/germline and 21 by tumor-only bioinformatic analysis. Affected genes are presented in order of frequency.

The 48 tumor and 27 blood DNA samples were sequenced successfully. After filtering all tumors showed one or more sequence variants, with an average of 4.6 (range: 1–13) per tumor and a total of 223 non-synonymous variants with effect on the amino acid sequence. Seventy of the 120 different genes analyzed were found affected in at least one tumor, 40 of which recurrently, in two or more tumors. Most frequently occurring variants included LRP1B, APC, NOTCH1, TSC2, KRAS, BRCA1 and NOTCH2 and ATM (Fig. 2). A case-by-case list of all variants is presented in Supplementary Table 2.

In 27 cases it was possible to compare the sequence variants in the tumor DNA to its corresponding germline DNA, allowing to distinguish somatic from germline variants. This analysis resulted in designating 78/136 (57%) variants as germline (yellow boxes in Fig. 2). As consequence, 7 of 27 (26%) cases did not harbor somatic sequence variants in the 120 genes analyzed. Conversely, seven variants that were discarded in the filtering of the single tumor data (allowing only those with < 1% presence in the normal population) were rescued when the germline match designated them as somatic (blue boxes in Fig. 2). In addition, analyzing tumor and germline sequencing data allowed analysis of copy number changes. We found four cases with high level gene amplifications of 6 copies or more in MET (20 copies), NRAS (10 copies), ATM (8 copies; this case also carried a nonsense mutation in ATM), and CTNNB1 (6 copies) (pink boxes in Fig. 2).

This makes the final result of 58 non-synonymous somatic variants in 33 different genes occurring in 20 of the 27 matched tumor/germline samples, with a range of 1–8 variants per tumor. In Supplementary Fig. 1 we marked these mutations as missense, nonsense or splice variants, showing for example that all mutations in APC and NF1 are nonsense, leading to truncated proteins. The most frequent somatically mutated gene is PIK3CA, observed in 5 tumors, followed by APC and ATM in 4, KRAS, NF1, LRP1B and BRCA1 in 3, and ERBB3, CTNNB1, NOTCH2 and CDKN2A in 2 tumors. Grouping mutated genes according to affected signaling pathways, we identified 7 cases with mutations in the MAPK/ERK pathway (2 NF1, 3 KRAS, 1 BRAF and 1 NF1/MAP2K1), 7 cases with mutations in the DNA repair pathway (3 ATM, 3 BRCA1 and 1 ATM/BRCA2), 6 cases with mutations in the WNT pathway (4 APC and 2 CTNNB1) and 6 cases with mutations in the PI3K pathway (4 PIK3CA, 1 PIK3CA/PIK3R2, 1 MTOR). The affected pathways did not occur in a mutually exclusive manner.

Table 3 presents a list of specific actionable mutations for which there is clinical and preclinical evidence for treatment, according to the expert-guided precision oncology knowledge database OncoKB classification (https://www.OncoKB.org)^36^. Five mutations in PIK3CA, one in BRCA1 and one in IDH1 found in this study are FDA-recognized biomarkers (level 1) and one ERBB2 mutation is a standard care biomarker (level 2A), predictive of response to FDA-approved drugs in specific tumor types. In addition, there is compelling evidence for one BRAF and two KRAS mutations as biomarkers of response to non-standard care drugs (level 3A). Finally, for three KRAS mutations, one CDKN2A and three NF1 mutations there is strong preclinical evidence for drug response in all solid tumors. These 16 mutations were found in 12 of the 27 patients (Supplementary Fig. 1).

### Correlations genetic and clinical data

Comparing the 7 cases without somatic variants with the 20 cases that had one or more, we found no differences in clinical variables as age, smoking or wood dust etiology, histological subtype, disease stage, recurrence or survival. We also did not observe any particular clinical features among those cases with the highest number of somatic variants.

Next we analyzed the clinical characteristics of the cases with mutations in one of the four described pathways. MAPK/ERK pathway mutations were seen only in papillary and colonic type ITACs (Fisher Exact Chi2, p = 0.026), while DNA repair, WNT and PI3K pathway mutations occurred in all histological subtypes. No significant correlations were found between any of the mutated pathways and age, tobacco smoking, tumor stage, recurrence or patient status. However, all PI3K pathway mutations occurred in stage I-II tumors (Fisher Exact Chi2, p = 0.284), while Wnt pathway mutations were more frequent in cases that remained disease-free during follow-up (Fisher Exact Chi2, p = 0.060) (Table 2). Finally, none of the four mutated pathways was related to overall or disease-specific survival. We did find a tendency toward longer disease-free survival in cases with mutations in WNT, MAPK/ERK and PI3K pathways, however, in multivariate logistic regression analysis including the prognostic factors tumor stage and histological subtype, none of the four mutated pathways came out as an independent prognostic factor (Supplementary Fig. 2 and Supplementary Table 1).

**Table 2:** Correlation between mutated pathways and clinical characteristics.

Pathway	MAPK/ERK			DNA repair			WNT			PI3K		
	wt	mut	P-value	wt	mut	P-value	wt	mut	P-value	wt	mut	P-value
**Histological type**												
pap/col	10	7		12	5		13	4		14	3	
sol/muc	10	0	0.026	7	3	1.000	8	2	1.000	7	3	0.638
**Tumor stage**												
I-II-III	15	6		15	6		16	5		15	6	
IVa-IVb	5	1	1.000	4	2	1.000	5	1	1.000	6	0	0.284
**Recurrence**												
No	8	4		9	3		7	5		9	3	
Yes	12	3	0.662	10	5	0.696	14	1	0.060	12	3	1.000
**Patient status**												
Alive	8	2		6	4		7	3		7	3	
dod	8	1		8	1		9	0		8	1	
doc	4	4	0.163	5	3	0.327	5	3	0.135	6	2	0.598
**Tobacco**												
No	10	3		9	4		10	3		11	2	
Yes	7	4	0.659	8	3	1.000	8	3	1.000	8	3	0.630

MAPK/ERK pathway genes include NF1, KRAS, BRAF and MAP2K1; DNA repair pathway genes include ATM, BRCA1, BRCA2 and DNMT3A; WNT pathway genes include APC and CTNNB1; PI3K pathway genes include PI3K, PIK3R2, AKT, MTOR; pap/col: papillary and colonic type ITAC; sol/muc: solid and mucinous type ITAC, DOD: died of disease, DOC: died of other causes.

## Discussion

Until recent years DNA sequencing was restricted to the field of basic cancer research and implementation in the clinical practice was hampered by the need for quality samples, the complexity of data processing, the difficulty of clinical interpretation of the genetic variants, and the cost and time of analysis. One approach to overcome these difficulties is to analyze exclusively those genes that are clinically relevant, using panels that represent genes frequently involved in one type of cancer or genes against which inhibitory drugs have been developed. These panels can also provide information useful for histopathological classification, and evaluation of prognosis. Currently, tumor-specific sequencing panels have been established as standard care in melanoma, breast, ovarian, colorectal and lung cancer^39,43–47^. Panels are also being used in head and neck cancer^48,49^ and in otolaryngology for genetic counseling unrelated to cancer^50^. The main advantages of panel sequencing in comparison to exome or whole genome sequencing are speed, cost, easier data interpretation and lower data storage requirements. Unlike other technologies such as microchips, panel sequencing also has great versatility and allows modification of genes that may gain or lose relevance over time. Furthermore, gene panels achieve a high coverage of sequencing (500X or more), resulting in a greater sensitivity and accuracy of mutation detection^39,51^.

In this retrospective study, we chose fresh frozen tumor samples for sequencing with a panel of 120 potentially actionable genes. Our analyses were successful in 100% of the samples and the average coverage was 300-500X. In the 48 tumors in this study, sequence variants were found in 70 different genes, 40 of which occurring in two or more tumors. However, in the 27 cases where we could co-analyze tumor and germline DNA, it became clear that only 43% of variants were somatic, in spite of the thorough filtering process in the tumor-only bioinformatic analysis (Supplementary data). In addition, 7 additional somatic variants that in the tumor-alone analysis had been filtered out were now classified as somatic. In sum, the 27 tumor/germline matched tumors carried a total of 58 somatic variants occurring in 33 different genes. The importance of co-analyzing germline DNA for the identification of true somatic actionable genetic alterations, present only in the tumor cells has been observed in similar studies^39,52^. The variants observed in NOTCH1, 2 and 3 illustrates this point: 11/27 (41%) cases showed alterations in one of these genes analyzing only the tumor DNA, however, only in three cases the variants appeared to be somatic when comparing tumor with germline DNA (Fig. 2).

Distinguishing somatic from germline variants is especially relevant with mutations of which the pathogenicity is not known or not clearly demonstrated. There are hotspot mutations, however, that are well-known pathogenic alterations, described in many different tumors and would not need to be confirmed by analysis of the corresponding germline DNA. In our series, for example KRAS was found mutated at the well-known hotspot exon 2 codon 12/13 site in 7 of the 48 tumor samples (Fig. 2). Of only 3 of these cases we had germline DNA available and indeed these showed absence of KRAS mutation. Other examples are hotspot CTNNB1 T41A and IDH1 R132C mutations. In addition, clearly deleterious variants such as stop-gain and frameshift mutations that lead to a truncated protein may be considered somatic without comparison to the germline DNA. In the series of 27 tumor/germline matched analyses, 4 cases harbored protein-truncating APC mutations and indeed all were observed only in the tumor sample.

This study is the first to apply NGS to screen for gene mutations in ITAC. We found recurrent somatic sequence variants in PIK3CA, APC, ATM, KRAS, NF1, LRP1B, BRCA1, ERBB3, CTNNB1, NOTCH2 and CDKN2A. There are few genetic studies on ITAC available to compare our data with. Only KRAS mutations have been studied extensively and yielded a frequency of 13–16%^9,22,25,27,28,30,32^ which is in alignment with our finding of 15% (7/48 cases). Two publications, however, have reported 43–50% KRAS mutations^26,29^. Mutations in BRAF appear to be infrequent in ITAC, ranging from 0–6%^25–28,32^, again in agreement with our current results (1/48 cases). Also truncating mutations in APC have been studied before in ITAC but none were found^29,30^, whereas our results showed 11/48 cases with mutations, 8 of which truncating. In the 27 tumor/germline matched cases 4 (15%) appeared as true somatic truncating APC mutations. TP53 mutations have been found in 40%-50% of ITAC^8,23,24^, however, as this gene is not clinically actionable, it was not included in our sequencing panel. Nevertheless, functional p53 status could serve as a predictive biomarker for response to chemotherapy^37^. In spite of the histological resemblance, ITAC do not have a mutational profile similar to colorectal adenocarcinoma. Although mutations in TP53, APC, CTNNB1, PIK3CA, KRAS, and BRAF occur in both tumors, the frequency is much lower in ITAC^8,23–28,32,53^. In fact, our data showed a low mutation frequency in general, the most frequent being PIK3CA, found in 19% of tumors. Perhaps this finding is related to the unique etiology of wood dust in ITAC. It may be speculated that ITAC do harbor frequent mutations, but in other genes than those included in our NGS panel, or that epigenetic events play an important role in ITAC. Future studies are needed to answer this question.

A large number of the somatic gene mutations affected PI3K, MAPK/ERK, WNT and DNA repair signaling pathways, although not in a mutually exclusive manner. Neither were specific mutated genes or pathways correlated to clinical factors as age or tobacco/wood dust etiology. In addition, with the exception of MAPK/ERK pathway mutations that all occurred in papillary and colonic type tumors, we were unable to find relevant differences between the four ITAC histological subtypes. This seems to suggest that ITAC is a genetically homogeneous group of tumors, which contrasts with previous claims that mucinous type ITAC stands apart from the other subtypes in terms of TP53 mutation and p53 overexpression, nuclear b-catenin and E-cadherin expression, gene promotor hypermethylation and chromosomal copy number alteration profile^22,35,53–55^. Finally, mutated pathways MAPK/ERK, DNA repair, WNT and PI3K did not correlate to tumor stage, overall or disease-specific survival. We did find a tendency for longer disease-free survival in cases with mutations in WNT, MAPK/ERK and PI3K pathways. However, with only 27 cases the statistical value of these correlations is very limited and studies of larger series of cases are necessary to be able to correlate mutated genes or pathways to clinical variables.

The main aim of this study was to identify actionable gene mutations that can guide the choice for new personalized target-specific therapies in ITAC patients. We found a total of 16 mutations in 12 cases that according to the expert-guided precision oncology knowledge database OncoKB classification (https://www.OncoKB.org)^36^, are biomarkers of FDA-approved targeted therapies or biomarkers with clinical and preclinical evidence as predictor of response (Table 3, Supplementary Table 1). Although it is clear that none of these anti-cancer drugs have been approved, let alone tested in ITAC patients, the eight patients with level 1 and 2A mutations in our series of 27 ITAC could benefit from targeted therapies that are FDA-approved and standard care in other cancer types. The fact that 7 of the 27 cases did not harbor mutations and that only 33 of the 120 analyzed genes were affected (Fig. 2) indicates that our panel needs adjustment. This can be done by adding newly discovered actionable genes, based on recent advances in the cancer genetics literature. Alternatively, as ITAC with its unique wood dust etiology apparently also carries a unique profile of genetic alterations, it may be valuable to first screen a series of cases using exome or whole genome NGS and perhaps also epigenetic analysis in order to compose a panel of genes specifically relevant to ITAC. We do believe that in terms of costs and data analysis, sequencing a limited set of genes with high coverage is optimal for clinical application.

**Table 3:** Level of clinical and preclinical evidence for treatment of specific actionable mutations, identified by OncoKB in the 27 ITACs.

Gene	Nr. Cases	Mutations	OncoKB level	Treatment Indications
*PIK3CA*	5	Q546R; K111F; H1047R; E726K; D939G	1	Apelisib + Fulvestrant in breast cancer
			2A	Fulvestrant + Buparlisib, Fulvestranst + Taselisib, Buparlisib, Copanlisib, GDC-0077, Serabelisib, Taselisib in Breast Cancer
*BRCA1*	1	P1603Rfs*13	1	Rucaparib, Niraparib in Peritoneal Serous Carcinoma and Ovarian Cancer
			2A	Olaparib in Peritoneus Serous Carcinoma and Ovarian Cancer
			2A	Talazoparib, Olaparib in Breast Cancer
*IDH1*	1	R132C	1	Ivosidenib in Acute Myeloid Leukemia
*ERBB2*	1	S310F	2A	Ado-Trastuzumab Emtasine in NSCLC
			3A	Neratinib in NSCLC and Breast Cancer
*BRAF*	1	D594N	3A	Cobimetinib in Histiocytosis
*KRAS*	3	G12D; G13D	3A	Cobimetinib in Histiocytosis
			4	Cobimetinib, Trametinib, Binimetinib in All Solid Tumors
			R1	Panitumumab, Cetuximab in Colorectal Cancer
*CDKN2A*	1	R58*	4	Ribociclib, Abemaciclib, Palbociclib in All Solid Tumors
*NF1*	3	R2237*; S2649*; T2423Nfs*4	4	Trametinib, Cobimetinib in All Solid Tumors

Nr. Cases: Number of ITAC tumors affected by these gene mutations; OncoKB refers to an expert-guided precision oncology knowledge database (https://www.OncoKB.org).^32^ OncoKB level 1: US Food and Drug Administration (FDA) approved drug for this tumor type; level 2A: Standard care biomarker predictive of response for this tumor type, not neccesarily FDA recognized; level 3A: Biomarker predictive of response for this tumor type but not yet applied in standard care; level 4: Preclinical evidence of biomarker as drug response predictor; R1: Standard care biomarker predictive of resistance to an FDA-approved drug in this tumor type.

In conclusion, this study was aimed at testing the usefulness of sequencing a dedicated panel of genes for the identification of clinically actionable gene mutations. An important finding is that additional sequencing analysis of the corresponding germline DNA is crucial for a thorough interpretation of somatic mutations. The 27 matched tumor/germline analyses indicated one or more potentially actionable somatic mutations in 20 cases (74%). In 8 cases (30%) we identified biomarkers of FDA-approved targeted therapies. Our data do not point to a specific subgroup of ITAC patients that carry actionable mutations; the 20 cases with one or more actionable mutations included all histological subtypes and tumor stages (Suppl Fig. 1), so in principle all newly diagnosed ITAC patients could possibly benefit from NGS testing to guide personalized treatment with specific inhibitor drugs. Such treatment should ideally be conducted in specifically designed next-generation sequencing clinical trials with molecularly guided recruitment in clinical referral centers or multi-institutional trials.

## Supplementary Information

Supplementary Information.

## Data Availability

All sequence data will be made accessible through public genomic repositories.
