# Supplementary material for: Next-generation sequencing for identification of actionable gene mutations in intestinal-type sinonasal adenocarcinoma
Source: Sci Rep. 2021 Jan 26;11:2247. doi: 10.1038/s41598-020-80242-z (PMC7838394; doi:10.1038/s41598-020-80242-z)
Supplement: Supplementary file 1 — Supplementary Information. [file 41598_2020_80242_MOESM1_ESM.docx]

**Supplementary data**

**­­­­­­­­­­­­­­­­­­­­­­­Next-generation sequencing for identification of actionable gene mutations in intestinal-type sinonasal adenocarcinoma**

Paula Sánchez-Fernández^1#^, Cristina Riobello^2#^, María Costales^1^, Blanca Vivanco^3^, Virginia N. Cabal^2^, Rocío García-Marín^2^, Laura Suárez-Fernández^2^, Fernando López^1^, Rubén Cabanillas^4^, Mario A. Hermsen^2^*, José Luis Llorente^1^

**Supplementary Figure 1.** Final list of somatic, actionable mutations in 27 ITACs in order of frequency.

**
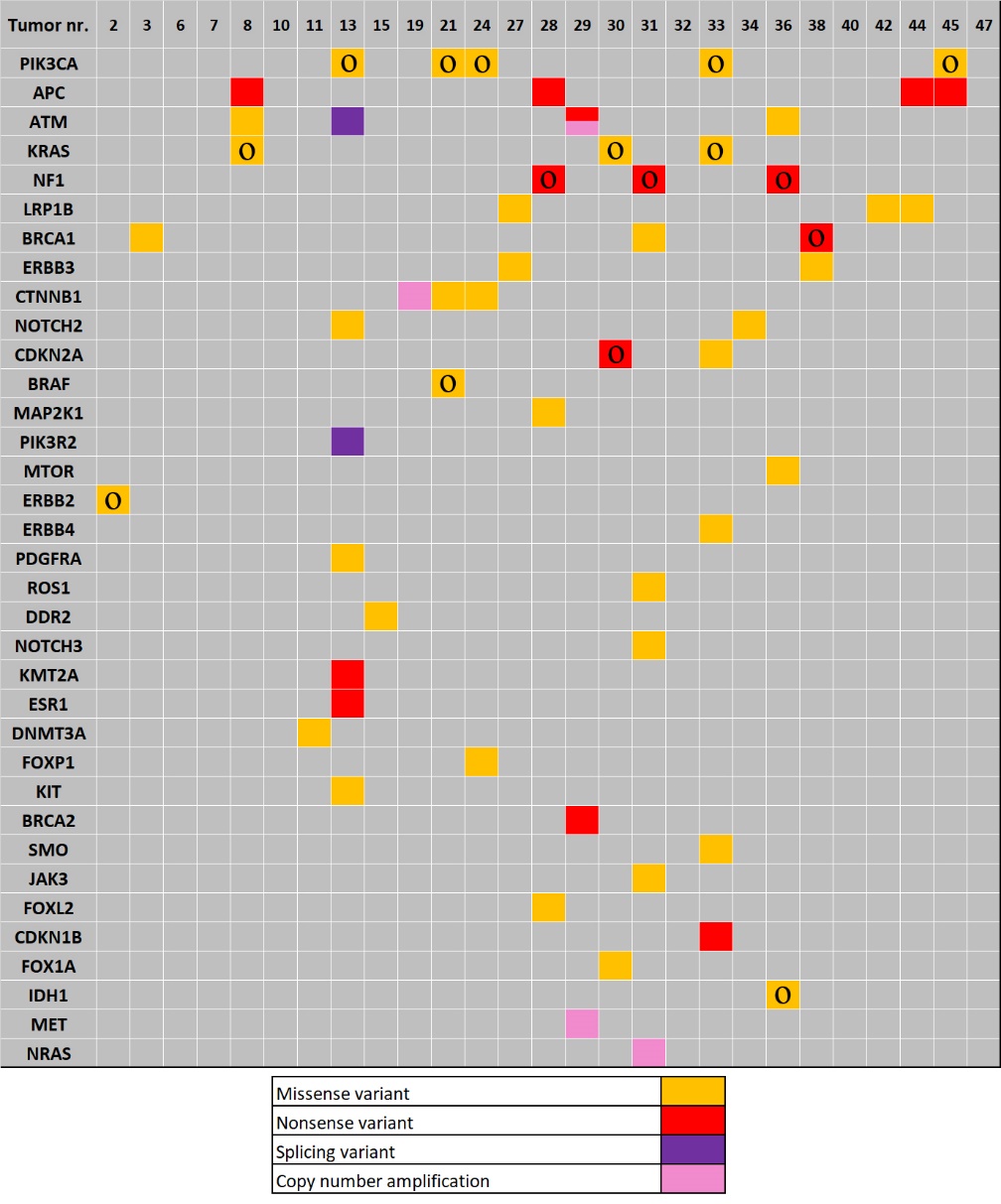
**

Legend: ''o'': mutations for which there is clinical and preclinical evidence for treatment, according to the expert-guided precision oncology knowledge database OncoKB classification (www.OncoKB.org).

**Supplementary Figure 2.** Disease-free survival according to mutated pathways MAPK/ERK (A), DNA repair (B), WNT (C) and PI3K (D).

**
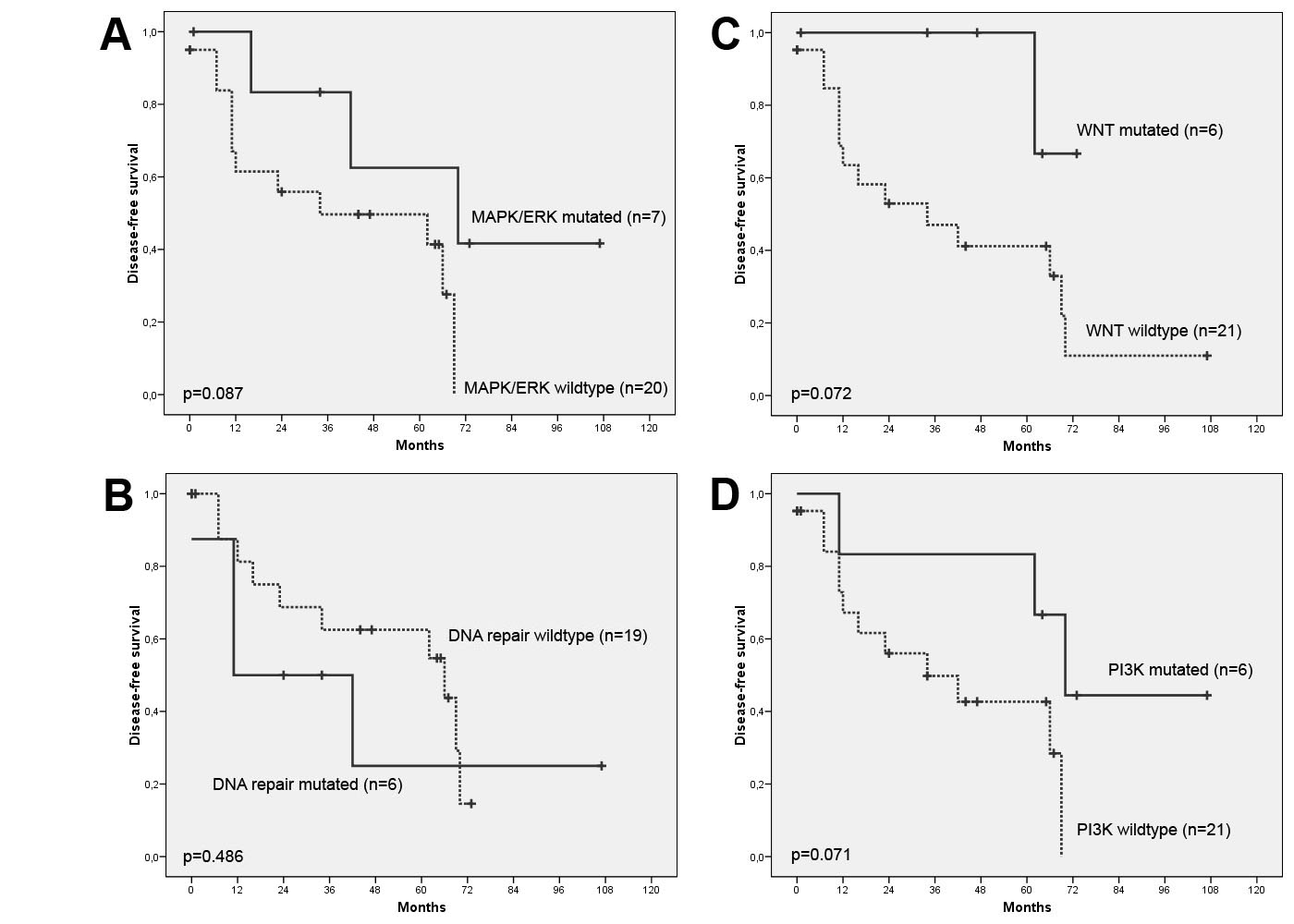
**

**Supplementary Table 1.** Multivariate multivariate logistic regression analysis of disease-free survival according to variabls tumor stage, histological type and mutated MAPK/ERK, DNA repair, WNT and PI3K pathways.

|  | Univariate | | Multivariate | |
| --- | --- | --- | --- | --- |
|  |  | Significance | Hazard ratio | Significance |
| Stage I-II-III vs IVa-b | Log rank | p=0.015 | 3.021 | p=0.194 |
| Pap-Col vs Sol-Muc | Log rank | p=0.202 | 0.484 | p=0.309 |
| MAPK/ERK pathway | Log rank | p=0.087 | 0.221 | p=0.187 |
| DNA repair pathway | Log rank | p=0.486 | 1.866 | p=0.360 |
| Wnt pathway | Log rank | p=0.072 | 0.320 | p=0.308 |
| Pi3K pathway | Log rank | p=0.071 | 0.913 | p=0.934 |

Supplementary Table 2A. All sequence variants identified in 27 ITAC by tumor/germline bioinformatic analysis.

| Tumor nr | Gene | DNA variant | Protein variant | Protein Effect | MAF | Zygosity |
| --- | --- | --- | --- | --- | --- | --- |
| 2 | ERBB2 | c.929C>T | p.S310F | NO_SYN | 0,101 | HETZ |
|  | NOTCH1 | c.5015G>A | p.R1672H | NO_SYN | 0,411 | HETZ |
|  | NOTCH1 | c.2542G>A | p.E848K | NO_SYN | 0,440 | HETZ |
|  | NOTCH3 | c.6097C>T | p.P2033S | NO_SYN | 0,503 | HETZ |
|  | MTOR | c.6097C>T | p.R1480C | NO_SYN | 0,073 | HETZ |
| 3 | BRCA1 | c.4663G>A | p.V1534M | NO_SYN | 0,765 | HOMZ_ALT ["LOH"] |
|  | MYD88 | c.16_34delGCTGAGGCTCCAGGACCGC | p.A6Pfs*39 | FRAMESHIFT | 0,436 | HETZ |
| 6 | MET | c.467C>T | p.S156L | NO_SYN | 0,488 | HETZ |
|  | ROS1 | c.1144T>C | p.Y382H | NO_SYN | 0,508 | HETZ |
|  | BRD4 | c.1445C>T | p.P482L | NO_SYN | 0,483 | HETZ |
|  | DDR2 | c.745C>T | p.H249Y | NO_SYN | 0,403 | HETZ |
|  | NOTCH1 | c.4691A>G | p.H1564R | NO_SYN | 0,547 | HETZ |
| 7 | NOTCH1 | c.2783C>T | p.T928M | NO_SYN | 0,430 | HETZ |
|  | KTM2A | c.1504G>A | p.E502K | NO_SYN | 0,484 | HETZ |
| 8 | APC | c.5826_5829delC | p.D1942Efs*27 | FRAMESHIFT | 0,389 | HETZ |
|  | ATM | c.3161C>G | p.P1054R | NO_SYN | 0,820 | HOMZ_ALT ["LOH"] |
|  | KRAS | c.38G>A | p.G13D | NO_SYN | 0,536 | HETZ |
|  | NOTCH2 | c.4238T>A | p.L1413H | NO_SYN | 0,468 | HETZ |
|  | APC | c.3949G>C | p.E1317Q | NO_SYN | 0,747 | HETZ |
|  | NF1 | c.7468G>C | p.V2490L | NO_SYN | 0,727 | HETZ |
|  | ESR1 | c.316A>G | p.S106G | NO_SYN | 0,492 | HETZ |
| 10 | CSF1R | c.1517A>C | p.H506P | NO_SYN | 0,485 | HETZ |
|  | TSC2 | c.1574A>G | p.N525S | NO_SYN | 0,462 | HETZ |
| 11 | DNMT3A | c.1895A>G | p.H632R | NO_SYN | 0,200 | HETZ |
|  | ERBB4 | c.882A>G | p.P294P | SPLICING | 0,495 | HETZ |
|  | IGFR1 | c.16G>A | p.G6R | NO_SYN | 0,466 | HETZ |
|  | PDGFRA | c.2365G>C | p.G789R | NO_SYN | 0,498 | HETZ |
|  | MLP | c.1771T>G | p.Y591D | NO_SYN | 0,049 | HETZ |
| 13 | PIK3CA | c.331A>G | p.K111E | NO_SYN | 0,252 | HETZ |
|  | PIK3CA | c.1361A>G | p.D454G | NO_SYN | 0,269 | HETZ |
|  | ATM | c.497-2A>T | - | SPLICING | 0,282 | HETZ |
|  | NOTCH2 | c.4733G>A | p.R1578H | NO_SYN | 0,274 | HETZ |
|  | PIK3R2 | c.1010+3A>T | - | SPLICING | 0,242 | HETZ |
|  | PDGFRA | c.1364A>G | p.K455R | SPLICING,NO_SYN | 0,282 | HETZ |
|  | KMT2A | c.472dupA | p.R158Kfs*12 | FRAMESHIFT | 0,257 | HETZ |
|  | KMT2A | c.7246G>T | p.E2419* | STOP_GAINED | 0,265 | HETZ |
|  | ESR1 | c.1057G>T | p.E353* | STOP_GAINED | 0,197 | HETZ |
|  | KIT | c.1039C>A | p.Q347K | NO_SYN | 0,236 | HETZ |
|  | BRCA1 | c.3331_3334delCAAG | p.Q1111Nfs*5 | FRAMESHIFT | 0,665 | HETZ |
|  | AR | c.2357T>C | p.V786A | NO_SYN | 0,491 | HETZ |
| 15 | DDR2 | c.1323G>A | p.M441I | NO_SYN | 0,767 | HOMZ_ALT ["LOH"] |
|  | LRP1B | c.7420G>A | p.G2474S | NO_SYN | 0,392 | HETZ |
|  | LRP1B | c.2005A>G | p.I669V | NO_SYN | 0,641 | HETZ |
|  | NOTCH1 | c.4028C>T | p.A1343V | NO_SXN | 0,053 | HETZ |
|  | MGMT | c.55C>T | p.L19F | NO_SYN | 0,636 | HETZ |
| 19 | ESR1 | c.352T>C | p.S118P | NO_SYN | 0,618 | HETZ |
|  | IGF2 | c.59C>T | p.S20L | NO_SYN | 0,335 | HETZ |
|  | CBL | c.1227+4C>T | - | SPLICING | 0,663 | HETZ |
|  | EWR1 | c.1276C>T | p.P426S | NO_SYN | 0,504 | HETZ |
|  | EWR1 | c.1408G>A | p.G470S | NO_SYN | 0,436 | HETZ |
|  | TSC1 | c.2814-1G>C | - | SPLICING | 0,067 | HETZ |
| 21 | PIK3CA | c.1637A>G | p.Q546R | NO_SYN | 0,295 | HETZ |
|  | CTNNB1 | c.1004A>T | p.K335I | NO_SYN | 0,600 | HETZ |
|  | BRAF | c.1780G>A | p.D594N | NO_SYN | 0,315 | HETZ |
|  | CSF1R | c.1517A>C | p.H506P | NO_SYN | 0,476 | HETZ |
| 24 | PIK3CA | c.2816A>G | p.D939G | NO_SYN | 0,255 | HETZ |
|  | CTNNB1 | c.133_135delTCT | p.S45del | INFRAME DEL | 0,505 | HETZ |
|  | FOXP1 | c.1144C>A | p.P382T | SPLICING,NO_SYN | 0,309 | HETZ |
|  | NOTCH2 | c.7075C>G | p.P2359A | NO_SYN | 0,517 | HETZ |
|  | LRP1B | c.6428-49A>C | - | INTRONIC | 0,529 | HETZ |
|  | LRP1B | c.3128C>G | p.T1043S | NO_SYN | 0,491 | HETZ |
|  | MDM2 | c.1492delT | p.*498del | FRAMESHIFT | 0,403 | HETZ |
|  | ALK | c.661G>C | p.G221R | NO_SYN | 0,518 | HETZ |
|  | BRCA2 | c.6100C>T | p.R2034C | NO_SYN | 0,496 | HETZ |
| 27 | LRP1B | c.2152C>A | p.H718N | NO_SYN | 0,297 | HETZ |
|  | LRP1B | c.10844G>C | p.G6315A | NO_SYN | 0,786 | HOMZ_ALT ["LOH"] |
|  | ERBB3 | c.217G>A | p.D73N | NO_SYN | 0,328 | HETZ |
|  | ATM | c.5558A>T | p.D1853V | NO_SYN | 0,245 | HETZ |
|  | TSC2 | c.1318G>A | p.G440S | NO_SYN | 0,288 | HETZ |
|  | MET | c.2975C>T | p.D1853V | NO_SYN | 0,245 | HETZ |
|  | FGFR2 | c.2305-216C>T | - | INTRONIC | 0,464 | HETZ |
|  | FGFR2 | c.170C>T | p.S57L | NO_SYN | 0,487 | HETZ |
| 28 | APC | c.4585C>T | p.Q1529* | STOP_GAINED | 0,405 | HETZ |
|  | NF1 | c.7946C>G | p.S2649* | STOP_GAINED | 0,423 | HETZ |
|  | MAP2K1 | c.622G>C | p.D208H | NO_SYN | 0,291 | HETZ |
|  | FOXL2 | c.695C>T | p.A232V | NO_SYN | 0,309 | HETZ |
|  | MAP2K4 | c.43G>A | p.G15S | NO_SYN | 0,275 | HETZ |
| 29 | ATM | c.1744T>C | p.F582L | NO_SYN | 0,956 | HOMZ_ALT ["LOH"] |
|  | BRCA2 | c.9976A>T | p.K3326* | STOP_GAINED | 0,900 | HOMZ_ALT ["LOH"] |
|  | LRP1B | c.2005A>G | p.I669V | NO_SYN | 0,448 | HETZ |
|  | TSC2 | c.5359G>A | p.G1787S | NO_SYN | 0,169 | HETZ |
| 30 | KRAS | c.35G>A | p.G12D | NO_SYN | 0,439 | HETZ |
|  | CDKN2A | c.172C>T | p.R58* | STOP_GAINED | 0,815 | HOMZ_ALT |
|  | FOX1A | c.442C>G | p.L148V | NO_SYN | 0,855 | HOMZ_ALT ["LOH"] |
|  | FOXP1 | c.1762G>A | p.A588T | NO_SYN | 0,494 | HETZ |
|  | APC | c.4336G>A | p.A1446T | NO_SYN | 0,353 | HETZ |
|  | ROS1 | c.1958C>T | p.S653F | NO_SYN | 0,365 | HETZ |
|  | HGF | c.1211C>A | p.S404Y | NO_SYN | 0,577 | HETZ |
|  | TSC1 | c.3103G>A | p.G1035S | NO_SYN | 0,502 | HETZ |
|  | LRP1B | c.7420G>A | p.G2474S | NO_SYN | 0,422 | HETZ |
| 31 | NF1 | c.6709C>T | p.R2237* | STOP_GAINED | 0,717 | HETZ |
|  | BRCA1 | c.4039A>G | p.R1347G | NO_SYN | 0,866 | HOMZ_ALT ["LOH"] |
|  | ROS1 | c.6116G>A | p.R2039H | NO_SYN | 0,223 | HETZ |
|  | NOTCH3 | c.3399C>A | p.H1133Q | NO_SYN | 0,861 | HOMZ_ALT ["LOH"] |
|  | JAK3 | c.2164G>A | p.V722I | NO_SYN | 0,864 | HOMZ_ALT ["LOH"] |
|  | RB1 | c.2652A>C | p.E884D | NO_SYN | 0,493 | HETZ |
| 32 | ATM | c.6067G>A | p.G2023R | NO_SYN | 0,485 | HETZ |
|  | CSF1R | c.2760G>C | p.E920D | NO_SYN | 0,503 | HETZ |
|  | CSF1R | c.2080G>A | p.E694K | NO_SYN | 0,505 | HETZ |
| 33 | PIK3CA | c.3140A>G | p.H1047R | NO_SYN | 0,306 | HETZ |
|  | KRAS | c.35G>A | p.G12D | NO_SYN | 0,278 | HETZ |
|  | CDKN2A | c.187G>C | p.Gly63Arg | NO_SYN | 0,782 | HOMZ_ALT ["LOH"] |
|  | ERBB4 | c.2008A>G | p.T670A | NO_SYN | 0,286 | HETZ |
|  | SMO | c.2285G>A | p.R762H | NO_SYN | 0,298 | HETZ |
|  | CDKN1B | c.376G>T | p.E126* | STOP_GAINED | 0,293 | HETZ |
|  | APC | c.1959G>A | p.R653R | SPLICING,SYN | 0,489 | HETZ |
|  | SMO | c.808G>A | p.V270I | NO_SYN | 0,498 | HETZ |
|  | SOCS1 | c.134_139dupTC | p.A47Vfs*168 | FRAMESHIFT | 0,388 | HETZ |
| 34 | NOTCH2 | c.4888C>T | p.R1630C | NO_SYN | 0,121 | HETZ |
|  | FOX1A | c.890C>T | p.P297L | NO_SYN | 0,523 | HETZ |
|  | FLT3 | c.*80A>G | - | 3'UTR | 0,511 | HETZ |
|  | KIT | c.1211C>T | p.A404V | NO_SYN | 0,561 | HETZ |
|  | FGFR1 | c.565C>T | p.R189C p.R189C | NO_SYN | 0,061 | HETZ |
| 36 | ATM | c.2051A>C | p.Q684P | NO_SYN | 0,436 | HETZ |
|  | NF1 | c.7267dupA | p.T2423Nfs*4 | FRAMESHIFT | 0,365 | HETZ |
|  | MTOR | c.6607G>A | p.G2203S | NO_SYN | 0,231 | HETZ |
|  | IDH1 | c.394C>T | p.R132C | NO_SYN | 0,294 | HETZ |
|  | ERBB3 | c.286A>G | p.T96A | NO_SYN | 0,514 | HETZ |
|  | PTCH1 | c.4081G>A | p.V1361M | NO_SYN | 0,514 | HETZ |
|  | ATR | c.3245G>A | p.R1082H | NO_SYN | 0,466 | HETZ |
|  | BRCA2 | c.2998A>C | p.I1000L | NO_SYN | 0,182 | HETZ |
|  | MITF | c.790G>A | p.A264T | NO_SYN | 0,492 | HETZ |
| 38 | BRCA1 | c.4807_4823del | p.P1603Rfs*13 | FRAMESHIFT | 0,569 | HETZ |
|  | ERBB3 | c.3529C>A | p.L1177I | NO_SYN | 0,926 | HOMZ_ALT |
| 40 | NOTCH1 | c.2542G>A | p.E848K | NO_SYN | 0,492 | HETZ |
|  | BCL2 | c.-373G>A | - | 5'UTR | 0,482 | HETZ |
|  | ROS1 | c.6061C>T | p.P2021S | NO_SYN | 0,493 | HETZ |
|  | NFKB2 | c.2249C>T | p.A750V | NO_SYN | 0,482 | HETZ |
|  | NTRK1 | c.16C>T | p.R6W | NO_SYN | 0,493 | HETZ |
|  | NTRK1 | c.53G>A | p.G18E | NO_SYN | 0,509 | HETZ |
| 42 | LRP1B | c.1604T>C | p.V535A | NO_SYN | 0,213 | HETZ |
|  | ATM | c.1229T>C | p.V410A | NO_SYN | 0,483 | HETZ |
| 44 | APC | c.4282G>T | p.G1428* | STOP_GAINED | 0,375 | HETZ |
|  | LRP1B | c.4496C>A | p.T1499K | NO_SYN | 0,176 | HETZ |
|  | CBL | c.2414_2416dup | p.L805_D806insV | INFRAME DUP | 0,372 | HETZ |
|  | FGFR3 | c.1354G>T | p.A452S | NO_SYN | 0,385 | HETZ |
| 45 | PIK3CA | c.2176G>A | p.E726K | NO_SYN | 0,267 | HETZ |
|  | APC | c.4618G>T | p.E1540* | STOP_GAINED | 0,422 | HETZ |
|  | NOTCH1 | c.5227G>A | p.A1743T | NO_SYN | 0,347 | HETZ |
|  | NTRK1 | c.575G>A | p.G192D | SPLICING,NO_SYN | 0,473 | HETZ |
|  | SH2B3 | c.464C>T | p.P155L | NO_SYN | 0,485 | HETZ |
| 47 | SH2B3 | c.639C>A | p.S213R | NO_SYN | 0,547 | HETZ |
|  | LRP1B | c.7452T>A | p.N2484K | NO_SYN | 0,496 | HETZ |

Legend: MAF: minor allele frequency.

Supplementary Table 2B. All sequence variants identified in 21 ITAC by tumor-only bioinformatic analysis.

| **Tumor nr** | **Gene** | **DNA variant** | **Protein variant** | **Protein Effect** | **MAF** | **Zygosity** |
| --- | --- | --- | --- | --- | --- | --- |
| 1 | LRP1B | c.2005A>G | p.I669V | NO_SYN | 0,511 | HETZ |
|  | ATM | c.146C>G | p.S49C | NO_SYN | 0,487 | HETZ |
|  | EPHA2 | c.2319delC | p.T774Pfs*37 | FRAMESHIFT | 0,274 | HETZ |
|  | FLT3 | c.1606C>T | p.P536S | NO_SYN | 0,475 | HETZ |
| 4 | NOTCH1 | c.4028C>T | p.A1343V | NO_SYN | 0,509 | HETZ |
|  | KRAS | c.35G>A | p.G12D | NO_SYN | 0,589 | HETZ |
| 5 | NRAS | c.553C>T | p.P185S | NO_SYN | 0,457 | HETZ |
|  | RB1 | c.1574C>G | p.A525G | NO_SYN | 0,693 | HETZ |
| 9 | APC | c.2413C>T | p.R805* | STOP_GAINED | 0,640 | HETZ |
|  | KRAS | c.176C>G | p.A59G | NO_SYN | 0,163 | HETZ |
| 12 | FOXP1 | c.1135G>A | p.A379T | NO_SYN | 0,490 | HETZ |
| 14 | NOTCH1 | c.1543G>C | p.E515Q | NO_SYN | 0,480 | HETZ |
|  | NOTCH2 | c.7223T>A | p.L2408H | NO_SYN | 0,244 | HETZ |
|  | LRP1B | c.12161A>C | p.E4054A | NO_SYN | 0,361 | HETZ |
|  | EPHA2 | c.830C>T | p.S277L | NO_SYN | 0,664 | HETZ |
|  | PDGFRA | c.39_44delTCTTCT | p.L14_L15del | INFRAME DEL | 0,111 | HETZ |
| 16 | ATM | c.998C>T | p.S333F | NO_SYN | 0,463 | HETZ |
|  | NF1 | c.278G>A | p.C93Y | NO_SYN | 0,121 | HETZ |
|  | NF1 | c.2686delG | p.D896Ifs*6 | FRAMESHIFT | 0,126 | HETZ |
|  | KMT2A | c.6563G>A | p.R2188Q | NO_SYN | 0,407 | HETZ |
|  | PTCH1 | c.1306G>A | p.D436N | NO_SYN | 0,479 | HETZ |
| 17 | BRCA1 | c.2521C>T | p.R841W | NO_SYN | 0,486 | HETZ |
|  | ROS1 | c.2411C>A | p.T804N | NO_SYN | 0,420 | HETZ |
|  | BRD4 | c.755C>G | p.P252R | NO_SYN | 0,620 | HETZ |
|  | AR | c.1424C>T | p.A475V | NO_SYN | 0,996 | HOMZ_ALT |
| 18 | BRCA1 | c.3331_3334delCAAG | p.Q1111Nfs*5 | FRAMESHIFT | 0,386 | HETZ |
|  | CSF1R | c.895G>A | p.A299T | NO_SYN | 0,493 | HETZ |
|  | TSC2 | c.5383C>T | p.R1795C | NO_SYN | 0,449 | HETZ |
|  | MET | c.504G>T | p.E168D | NO_SYN | 0,472 | HETZ |
|  | EPHA2 | c.2875G>A | p.A959T | NO_SYN | 0,443 | HETZ |
|  | AKT1 | c.138C>A | p.D46E | NO_SYN | 0,470 | HETZ |
| 20 | APC | c.4088A>G | p.K1363R | NO_SYN | 0,744 | HETZ |
|  | BCRA2 | c.4419C>A | p.N1473K | NO_SYN | 0,129 | HETZ |
|  | BCRA2 | c.4421A>T | p.K1474I | NO_SYN | 0,130 | HETZ |
|  | SMO | c.808G>A | p.V270I | NO_SYN | 0,483 | HETZ |
|  | PTCH1 | c.3487G>A | p.G1163S | NO_SYN | 0,468 | HETZ |
| 22 | ATM | c.2932T>C | p.S978P | NO_SYN | 0,501 | HETZ |
|  | KRAS | c.35G>T | p.G12V | NO_SYN | 0,027 | HETZ |
|  | JAK3 | c.2152G>C | p.V718L | NO_SYN | 0,474 | HETZ |
| 23 | APC | c.1779G>A | p.W593* | STOP_GAINED | 0,398 | HETZ |
|  | BRCA1 | c.4039A>G | p.R1347G | NO_SYN | 0,268 | HETZ |
|  | BRCA1 | c.3284A>C | p.K1095T | NO_SYN | 0,282 | HETZ |
|  | ERBB2 | c.1960A>G | p.I654V | NO_SYN | 0,711 | HETZ |
|  | FLT3 | c.1774G>A | p.V592I | NO_SYN | 0,158 | HETZ |
|  | MTOR | c.4453G>A | p.E1485K | NO_SYN | 0,056 | HETZ |
|  | JAK3 | c.2164G>A | p.V722I | NO_SYN | 0,461 | HETZ |
| 25 | MAP2K2 | c.813C>G | p.D271E | NO_SYN | 0,381 | HETZ |
|  | LRP1B | c.13114A>T | p.N4372Y | NO_SYN | 0,482 | HETZ |
|  | LRP1B | c.11227G>A | p.G3743S | NO_SYN | 0,510 | HETZ |
|  | APC | c.933G>A | p.K311K | SPLICING,SYN | 0,543 | HETZ |
| 26 | TSC2 | c.5378G>A | p.R1793Q | NO_SYN | 0,514 | HETZ |
|  | KRAS | c.35G>A | p.G12D | NO_SYN | 0,284 | HETZ |
|  | LRP1B | c.12523C>A | p.P4175T | NO_SYN | 0,236 | HETZ |
|  | EPHA2 | c.2162G>A | p.R721Q | NO_SYN | 0,473 | HETZ |
| 35 | ERBB3 | c.2150C>T | p.S717L | NO_SYN | 0,4594 | HETZ |
|  | TSC2 | c.5116C>T | p.R1706C | NO_SYN | 0,53248 | HETZ |
|  | APC | c.1746dupA | p.S583Ifs*19 | SPLICING,FRAMESHIFT | 0,58484 | HETZ |
|  | ATR | c.2290A>G | p.K764E | NO_SYN | 0,64808 | HETZ |
|  | PALB2 | c.2816T>G | p.L939W | NO_SYN | 0,60027 | HETZ |
| 37 | ATM | c.1810C>T | p.P604S | NO_SYN | 0,48227 | HETZ |
|  | ATM | c.4388T>G | p.F1463C | NO_SYN | 0,4826 | HETZ |
|  | ERBB3 | c.1106A>G | p.N369S | NO_SYN | 0,67316 | HETZ |
|  | TSC2 | c.1574A>G | p.N525S | NO_SYN | 0,62824 | HETZ |
|  | ESR1 | c.-30C>T | - | 5'UTR | 0,87375 | HOMZ_ALT |
|  | MGMT | c.322G>A | p.E108K | NO_SYN | 0,62992 | HETZ |
|  | NTRK1 | c.16C>T | p.R6W | NO_SYN | 0,66763 | HETZ |
|  | LRP1B | c.12003G>T | p.W4001C | NO_SYN | 0,29799 | HETZ |
|  | BRD4 | c.3536A>G | p.K1179R | NO_SYN | 0,29799 | HETZ |
| 39 | NOTCH2 | c.7075C>G | p.P2359A | NO_SYN | 0,49922 | HETZ |
|  | KMT2A | c.1948A>G | p.N650D | NO_SYN | 0,44625 | HETZ |
|  | KMT2A | c.10648G>A | p.G3550R | NO_SYN | 0,48506 | HETZ |
|  | NRTK1 | c.2339G>A | p.R780Q | NO_SYN | 0,50712 | HETZ |
|  | FOXP1 | c.643C>G | p.P215A | NO_SYN | 0,50674 | HETZ |
|  | ERBB2 | c.2033G>A | p.R678Q | NO_SYN | 0,98049 | HOMZ_ALT |
|  | ERBB2 | c.1157C>A | p.A386D | NO_SYN | 0,98032 | HOMZ_ALT |
|  | NOTCH3 | c.6532C>T | p.P2178S | NO_SYN | 0,49342 | HETZ |
| 41 | BRCA1 | c.1367T>C | p.I456T | NO_SYN | 0,49156 | HETZ |
|  | TSC2 | c.5383C>T | p.R1795C | NO_SYN | 0,51685 | HETZ |
| 43 | IL7R | c.760G>A | p.A254T | NO_SYN | 0,484 | HETZ |
|  | TSC2 | c.1577G>A | p.S526N | NO_SYN | 0,496 | HETZ |
|  | CSF3R | c.2422G>A | p.E808K | NO_SYN | 0,495 | HETZ |
|  | MTOR | c.985G>A | p.A329T | NO_SYN | 0,469 | HETZ |
| 46 | NOTCH1 | c.4348G>A | p.E1450K | NO_SYN | 0,586 | HETZ |
|  | BRCA2 | c.430G>T | p.V144F | NO_SYN | 0,245 | HETZ |
|  | BRCA2 | c.5299A>T | p.K1767* | STOP_GAINED | 0,419 | HETZ |
|  | EPHA2 | c.334G>A | p.A112T | NO_SYN | 0,520 | HETZ |
| 48 | MLP | c.1102G>T | p.V368L | NO_SYN | 0,605 | HETZ |

Legend: MAF: minor allele frequency.

**Supplementary methods**

**Bioinformatic analysis of WES data**

WES results were processed using the bioinformatics software HD Genome One (DREAMgenics, Oviedo, Spain), certified with IVD/CE-marking. The exome analysis pipeline included the following steps:

*Quality control and Alignment*

Quality controls were performed using FastQC (http://www.bioinformatics.babraham.ac.uk/projects/fastqc/). Removal of low quality bases, adapters and other technical sequences was conducted with Trimmomatic.^1^ BWA-mem was then employed for alignment to the human reference genome (GRCh37/hg19), generating sorted BAM Files with SAMtools.^2^ Finally, optical and PCR duplicates were eliminated using Picard (http://broadinstitute.github.io/picard/).

*Somatic Variant Calling*

A variation of Sidrón algorithm was employed for the identification^3^ of SNVs and indels, which performance has been previously described.^4^ Somatic variants were identified using the following parameters: total read depth ≥15, variant frequency ≥0.05, base quality ≥20, mapping quality ≥30. Mutations detected in the tumor samples were interrogated in the normal sample in order to define the somatic status of each variant.

*CNV and CN-LOH identification*

The detection of CNVs and CN-LOH was performed employing exome2cnv, evaluating read depth and allelic imbalance computations. The algorithm employs a background of pooled samples processed using the same capturing protocol and sequencing technology.^51-52^

**References**

1. Bolger, A.M.; Lohse, M.; Usadel, B. Trimmomatic: Aflexible trimmer for Illumina sequence data. Bioinformatics 2014, 30, 2114–2120.

2. Li, H.; Handsaker, B.; Wysoker, A.; Fennell, T.; Ruan, J.; Homer, N.; Marth, G.; Abecasis, G.; Durbin, R.; Genome Project Data Processing, S. The Sequence Alignment/Map format and SAMtools. Bioinformatics 2009, 25, 2078–2079.

3. Puente, X.S.; Pinyol, M.; Quesada, V.; Conde, L.; Ordonez, G.R.; Villamor, N.; Escaramis, G.; Jares, P.; Bea, S.; Gonzalez-Diaz, M.; et al. Whole-genome sequencing identifies recurrent mutations in chronic lymphocytic leukaemia. Nature 2011, 475, 101–105.

4. Cabanillas, R.; Dineiro, M.; Castillo, D.; Pruneda, P.C.; Penas, C.; Cifuentes, G.A.; de Vicente, A.; Duran, N.S.; Alvarez, R.; Ordonez, G.R.; et al. A novel molecular diagnostics platform for somatic and germline precision oncology. Mol. Genet. Genomic Med. 2017, 5, 336–359.
